# Morbid Obesity and Thyroid Cancer Rate. A Review of Literature

**DOI:** 10.3390/jcm10091894

**Published:** 2021-04-27

**Authors:** Stefania Masone, Nunzio Velotti, Silvia Savastano, Emanuele Filice, Rossana Serao, Antonio Vitiello, Giovanna Berardi, Vincenzo Schiavone, Mario Musella

**Affiliations:** 1 Department of Clinical Medicine and Surgery, University of Naples “Federico II”, Via Pansini n. 5, 80131 Naples, Italy; silvia.savastano@unina.it (S.S.); emanuele.filice888@gmail.com (E.F.); 2 Department of Advanced Biomedical Sciences, University of Naples “Federico II”, Via Pansini n. 5, 80131 Naples, Italy; nunzio.velotti@gmail.com (N.V.); rossyserao2@gmail.com (R.S.); AntonioVitiello_@hotmail.it (A.V.); giovannaberardi88@gmail.com (G.B.); vincenzoschiavone92@gmail.com (V.S.); mario.musella@unina.it (M.M.)

**Keywords:** thyroid cancer, obesity, chronic inflammation, adipokines

## Abstract

In the past three decades, several recent studies have analyzed the alarming increase of obesity worldwide, and it has been well established that the risk of many types of malignancies is increased in obese individuals; in the same period, thyroid cancer has become the fastest growing cancer of all malignancies. We investigated the current literature to underline the presence of a connection between excess body weight or Body Mass Index (BMI) and risk of thyroid cancer. Previous studies stated that the contraposition between adipocytes and adipose-resident immune cells enhances immune cell production of multiple pro-inflammatory factors with subsequent induction of hyperlipidemia and vascular injury; these factors are all associated with oxidative stress and cancer development and/or progression. Moreover, recent studies made clear the mitogenic and tumorigenic action of insulin, carried out through the stimulation of mitogen-activated protein kinase (MAPK) and phosphoinositide-3 kinase/AKT (PI3K/AKT) pathways, which is correlated to the hyperinsulinemia and hyperglycemia found in obese population. Our findings suggest that obesity and excess body weight are related to an increased risk of thyroid cancer and that the mechanisms that combine overweight with this cancer should be searched for in the adipokine pathways and chronic inflammation onset.

## 1. Introduction

In the past three decades, several recent studies have analyzed the alarming increase of obesity worldwide and its role as a risk factor for the onset of different metabolic disorders, such as type 2 diabetes and cardiovascular diseases [1]. Moreover, it has been well established that the risk of many types of malignancies is increased in morbid obese individuals with a body mass index (BMI) > 40 kg/m^2^ or >35 kg/m^2^ in the presence of obesity-related comorbidities [2]. In the same period, thyroid cancer has become the fastest growing cancer of all malignancies, with an estimated 62,450 new cases in the United States and 52,900 new thyroid cancers developed in Europe [3].

Molecular mechanisms linking excessive adiposity with the development of thyroid cancer are complex and still not completely known; furthermore, the results of several observational studies have been conflicting or inconclusive [4,5].

This review is aimed to present the current knowledge of the connection between excess body weight BMI and the risk of thyroid cancer.

## 2. Thyroid Cancer

Thyroid cancer accounts for only 1% of solid organ malignancies and 0.5% of all cancer deaths. Although thyroid cancer is more common overall in females, men are twice as likely as women to die from this cancer [6]. Thyroid cancers exhibit a broad range of clinical behavior that varies from indolent tumors with low mortality in most cases, to very aggressive malignancies, for example, anaplastic thyroid cancer; differentiated forms, which represent 95% of cases, are the most common category of thyroid cancer and include papillary thyroid cancer (PTC), follicular thyroid cancer (FTC), and Hurthle cell thyroid cancer [7]. Papillary thyroid cancer is the most common type with the best overall prognosis. On the other hand, follicular thyroid cancer, Hurthle cell thyroid cancer, and poorly differentiated thyroid cancers are high-risk tumors characterized by hematogenous metastasis to distant sites (lungs and bones) [8]. PTC is the most common endocrine malignancy, accounting for 96.0% of total new endocrine cancers and 66.8% of deaths due to endocrine cancers [9].

Findings from DNA sequencing studies of thyroid cancer have revealed the genetic basis for most thyroid cancers: non-overlapping mutations of the RET, TRKA, RAS, and BRAF genes are found in about 70% of PTCs, and it is well known that these genes encode activators of the mitogen-activated protein kinase (MAPK) cascade [10]. The most frequent mutation in non-medullary thyroid cancer is the BRAF^T1799A^ mutation, which is exclusive to papillary thyroid cancer and papillary-thyroid-cancer-derived anaplastic thyroid cancer. Similarly, mutations in the RAS family of oncogenes also occur most frequently in follicular thyroid cancer and follicular-variant papillary thyroid cancer [11].

Considering the role of inflammation and immune response in the onset of thyroid cancer, a link between this tumor, in particular the PTC histotype, and autoimmune thyroid diseases, such as Hashimoto’s thyroiditis and Grave’s disease, has already been demonstrated, although the precise mechanism is still unclear [12]. It has been proposed that inflammation might facilitate the rearrangement of the RET/PTC genomic complex through the production of free radicals, cytokine secretion, and cellular proliferation [13]. In detail, it is possible that cytokines and chemokines released by the inflammatory tumoral stroma determine the survival of those thyroid cells in which RET/PTC rearrangements randomly occur, promoting the selection of clones that acquire additional genetic lesions and thus become resistant to oncogene-induced apoptosis [14].

Another consideration regards the evidence of immune-inflammatory cell infiltrates in thyroid cancer: carcinomas are often present with a remarkable lymphocytic infiltrate in the absence of the typical signs of autoimmune thyroiditis. This phenomenon is characterized by a lymphocytic infiltrate and is generally significantly higher in patients with PTC than in patients with benign lesions [15]. Moreover, Ryder et al. found that an infiltration of macrophages and immature dendritic cells in PTC is correlated with capsule invasion and extrathyroidal extension [16].

Described findings suggest that the interaction between inflammation and genetic factors can represent a fundamental moment in the alteration of the molecular mechanisms that drive thyroid tumorigenesis.

## 3. Obesity, Inflammation and Cancer Development

Obesity is a chronic, low-grade inflammatory, and non-transmissible disease that affects all ages with a worldwide prevalence of 13% (11% of men and 15% of women). More than 1.9 billion adults aged 18 years and older were overweight and of these over 650 million adults were obese. In 2016, 39% of adults aged 18 years and over (39% of men and 40% of women) were overweight. The worldwide prevalence of obesity nearly tripled between 1975 and 2016 [17]. Comorbidities in patients with obesity may be due to extra body weight on the musculoskeletal system or by increased secretion of free fatty acids, peptides, and adipocytokines produced by adipocytes. More common comorbidities include depression; biliary lithiasis; hepatic steatosis; dyslipidemia; arterial hypertension; coagulopathies; endothelial dysfunction; thyroid alterations; type 2 diabetes mellitus; polycystic ovarian syndrome and hypogonadism; breast, esophagus, pancreas, and colon neoplasms; and sleep apnea [18,19].

As the definition of obesity can vary between different countries, the WHO stated a standard classification according to patients’ BMI: 18.5–24.9 healthy weight; >25.0 overweight; >30 obese; >40 morbid obese [20].

Obesity is often accompanied by a low-grade chronic inflammatory state characterized by an increase in systemic markers of inflammation with a non-specific activation of the immune system, which is believed to contribute to the development of these obesity-associated pathologies [21].

In detail, contraposition between adipocytes and adipose-resident immune cells enhance immune cell production of multiple pro-inflammatory factors with subsequent induction of insulin resistance and hyperinsulinemia, hyperglycemia, hyperlipidemia, and vascular injury; these factors are all associated with oxidative stress and cancer development and/or progression [22,23].

This hypothesis is also supported by the observation that chronic infections are associated with 18% of cancer cases worldwide, as in the well-known mechanics of Helicobacter pylori infection role in gastric cancer onset, in which the intervention of immune response in the course of chronic infection determines the production of inflammatory cell mediators that sustain proliferative signaling, induce cell migration and metastasis, and promote angiogenesis [24].

For the first time in 1863, Virchow linked chronic inflammation and cancer development, observing an abundance of leukocytes in neoplastic tissue [25]. Analyzing mechanisms leading to different tumors, such as breast and colorectal cancer [26,27], it is nowadays clear that chronic inflammation produces the activation and transcription of factors such as nuclear factor kappa-light-chain-enhancer of activated B cells (NF-κB), STAT3, and activator protein 1 in pre-malignant cells; at the same time, obese white adipose tissue tends to have an increase in the production of leptin, which is pro-inflammatory, pro-angiogenic, and pro-proliferative, and a decrease in adiponectin, which is anti-inflammatory, anti-angiogenic, and anti-proliferative [28]. Moreover, obesity leads to increased endoplasmic reticulum stress, resulting in the activation of the unfolded protein response, with an increased oxidative stress, and in turn the upregulation of inflammatory cytokines [29]. All these pathways enhance cell proliferation and survival and promotes angiogenesis in conjunction with hypoxia [30].

Finally, recent studies have focused attention on the role of obese white adipose tissue in the activation of the inflammasome: multiprotein complexes that activate IL-1β and IL-18 pathways in response to pathogen-associated molecular patterns (PAMPs) and danger-associated molecular patterns (DAMPs) [31]. Inflammasomes have been shown to play a complex role in cancer through IL-1β, which promotes proliferation and invasion of tumors, and although there is no evidence for the role of inflammasome activation in obesity-associated cancer, it has been demonstrated that the inflammatory effect of leptin is dependent on IL-1β [32,33].

In such a scenario, recent evidence, primarily from bariatric surgery studies, indicates that substantial weight loss reduces cancer risk, most likely by attenuating adipose-related inflammatory mechanisms that can regulate tumor development and progression [34].

## 4. Obesity and Thyroid Cancer

Recent studies and metanalyses have investigated the relationship between obesity and the development of thyroid cancer.

Zhao et al. [35] first studied this field with a review of seven cohort studies for a total of 5154 thyroid cancer cases; the pooled results demonstrated that there was a statistically significant association between BMI and cancer risk. Authors also performed a stratified analysis according to sex, finding a statistically more significant association between BMI and thyroid cancer risk for males than for women.

Schmid and colleagues [36], pooling together data from 21 studies and 12,199 thyroid cancer cases, found a statistically significant 25% greater risk of thyroid cancer in overweight individuals and a 55% greater thyroid cancer risk in obese individuals; their analysis revealed that an increase of 5-unit in body mass index (BMI), 5 kg in weight, 5 cm in waist or hip circumference, and 0.1-unit in waist-to-hip ratio were associated with 30%, 5%, 5%, and 14% greater risks of thyroid cancer, respectively. Authors also evaluated histologic type, demonstrating that obesity was significantly positively related to papillary, follicular, and anaplastic thyroid cancers, whereas there was an inverse association with medullary thyroid cancer. Another important aspect is represented by the relation between obesity and the risk of cancer progression. Recently, Wang et al. [37] analyzed data from 1579 patients with PTC, clustering sample size based on BMI: underweight patients (BMI < 18.5 kg/m^2^), normal body patients (18.5 < BMI < 24.0 kg/m^2^), overweight patients (24.0 < BMI < 28.0 kg/m^2^), and obese patients (BMI > 28.0 kg/m^2^). They found a higher risk for extrathyroidal extension, advanced T stage (T III/IV), and advanced tumor-node-metastasis stage (TNM III/IV) in the overweight and obese patients’ groups, concluding that obesity is closely related to the risk of PTC and, particularly, that BMI is positively associated with the invasiveness of PTC.

The complex interplay among genetic variants of thyroid cancer and dietary intake has been recently investigated [38]: it is now clear that carbohydrate intake, such as alcohol and coffee consumption, are positively associated with thyroid cancer. Considering a stratification based on demographic characteristics, Ma et al. [39] presented a metanalysis on 32 studies in which they assessed that obese women have a higher risk of thyroid cancer onset when compared with obese men (RR = 1.43 vs. RR = 1.26); moreover, significantly elevated risk was observed in obese Caucasians and Asians and in the obese population >50 years-old as opposed to the young obese population (RR = 1.28 vs. RR = 1.23).

It is worth mentioning, on the other hand, that the results published in some recent studies reveal ongoing debate over the relationship between thyroid cancer and obesity. Rotondi retrospectively analyzed 4849 fine-needle aspiration cytology (FNAC) for thyroid nodules, concluding that a significant lower rate of Thy4/5 was observed in female obese patients [40]; Farfel et al. [41], on 760 incidence cases of thyroid cancer, proposed a multivariate analysis that demonstrated that BMI was not associated with cancer incidence.

## 5. Studies in Animal Models

Animal studies have elucidated the complex role between obesity and thyroid cancer, helping to interpret the pathogenetic mechanisms behind this complex correlation. Kim et al. [42] evaluated the role of diet-induced obesity on the development of thyroid cancer in a mouse model that spontaneously develops thyroid cancer, finding that a high-fat diet (HFD) increases thyroid tumor cell proliferation by increasing the protein levels of cyclin D1, phosphorylated retinoblastoma protein, serum leptin levels, and STAT3 target gene expression. The same results were found by Park [43], who demonstrated the effect of S3I-201, an inhibitor of STAT3 activity, on HFD-induced thyroid cancer progression in a murine model; the authors found decreased protein levels of cyclins D1 and B1, cyclin dependent kinase 4 (CDK4), CDK6, and phosphorylated retinoblastoma protein led to the inhibition of tumor cell proliferation.

## 6. Molecular Mechanisms

The role of Adiponectin (APN) in endocrine cancer risk has been widely studied, but there are still controversies on the exact mechanics that rule their anti-neoplastic function: it has been proposed that low APN levels could be associated with cancers due to an excess of fat mass and sex-steroid hormones with high levels of inflammation, but there is a lack of evidence on the pathway involved.

As stated by previous research, APN’s protective role on endocrine cancer cells is the activation of adenosine monophosphate-activated protein kinase (AMPK), which negatively influences cancer cells growth through p53 and p21- induced apoptosis [44,45]; moreover, it has been demonstrated that APN is able to down-regulate leptin-induced STAT3 phosphorylation, reducing tumor cell growth [46].

Despite some discrepancies, scientific research has amply demonstrated an inverse correlation between APN levels and the risk of endocrine neoplasms [47]. Considering that obesity frequently results in hypoadiponectinemia, it is very likely that the increased risk of cancer found in obese patients is at least partially attributable to the loss of the immunosuppressive effects induced by this hormone. Indeed, APN exerts its tumor suppressor effect both directly, through the interaction with its specific receptors, and indirectly, through the regulation of the immune response, angiogenesis, and insulin sensitivity. Through the activation of its receptors and the consequent activation of the adenosine monophosphate-activated protein kinase (AMPK), APN is able to determine both a reduction of the anabolic and proliferative pathways and an increased expression of important factors involved in the arrest of cell cycle and apoptosis, such as p21 and p53 [45]. Furthermore, AMPK activation determines an indirect inhibition of both MAPK and PI3K/AKTt/mTOR pathways, with an effect of downregulation of cell proliferation [48]. Regarding indirect anti-neoplastic effects, it has been shown that APN levels are inversely related to the degree of insulin resistance and to insulin levels [49]. Furthermore, the APN effect on insulin signaling seems to be also present at the post-receptor level. In addition, through interaction with the NF-kB pathway and its capacity of inhibiting myelomonocytic progenitor’s growth and macrophage phagocytic activity, APN is able to exert a real anti-inflammatory and immunomodulating effect [50]. Finally, it seems that APN may play a key role in angiogenesis regulation, but the studies currently available have provided conflicting results. In fact, some studies have shown that this adipokine is able to cause a conspicuous reduction in angiogenesis, while other studies conducted on mouse models of breast cancer suggest that adiponectin has a powerful pro-angiogenic effect [51]. Regarding the link between APN and thyroid cancer, some studies have shown an inverse relationship between APN levels and thyroid cancer [52]; however, further studies are required to confirm these findings.

Considering thyroid cancer, Mitsiades et al. [53] found lower levels of circulating APN in patients with any form of thyroid cancer compared to the control population; similarly, Warakomski et al., in a large prospective study, found that upper tertiles of IL-6 and leptin were associated with a higher clinical stage of PTC. The same results were found in the multicenter “EPIC” study from Dossus et al. [52] on 475 primary thyroid cancer cases: the authors underlined that adiponectin was inversely associated with cancer risk among women, whereas a positive association was revealed with IL-10. Supporting this interesting hypothesis, Cheng et al. [54] have shown that papillary thyroid carcinoma cell lines express a significantly lower number of AdipoR1 and AdipoR2 receptors than normal thyrocytes.

At the same time, APN may also express its anti-neoplastic role through insulin-sensitizing and angiogenesis-related effects. Considering that APN levels are inversely related to fasting insulin concentrations due to its significant effect on insulin post-receptor signaling, and considering that insulin supports tumor cell proliferation, it was proposed that APN is able to reduce tumor cell growth induced by insulin pathway [55,56].

Recently, some studies also highlighted the pro-carcinogenic role of leptin: Hedayati et al. [57] found Leptin levels were higher in thyroid cancer patients compared to healthy subjects, and Uddin and colleagues [58] demonstrated that leptin acts via its receptor to induce PTC cell proliferation and inhibit apoptosis.

**Figure 1:**
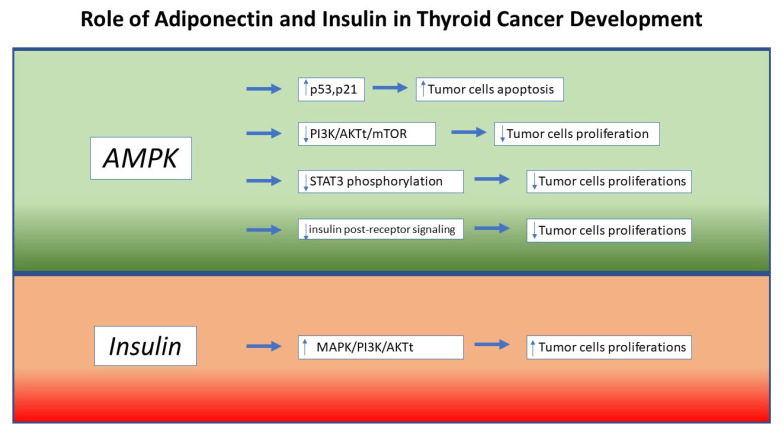
Molecular Mechanisms supporting the relation between obesity and thyroid cancer onset. AMPK: 5’ AMP-activated protein kinase; MAPK: mitogen-activated protein kinase; PI3K: Phosphoinositide 3-kinases; mTOR: mammalian target of rapamycin; STAT3: Signal transducer and activator of transcription 3; AKT: serine/threonine kinase.

Finally, it has been shown that the state of chronic hyperinsulinemia is associated with the development of different types of malignancies, such as lung, prostate, and breast cancer [59,60]. The mitogenic and tumorigenic action of insulin would seem to be carried out through the stimulation of mitogen-activated protein kinase (MAPK) and phosphoinositide-3 kinase/AKT (PI3K/AKT) pathways [61]. Of interest, several indirect mechanisms seem to underlie the link between insulin resistance and thyroid cancer. First, the condition of hyperinsulinemia and insulin resistance, typical of subjects with obesity, is associated with an increase in TSH levels, with a consequent enhancement of thyrocyte proliferation [62]. The increase in thyrocyte proliferation could lead to a mutational accumulation capable of triggering neoplastic transformation. The increase of TSH levels in the obese population probably represents an adaptive response aimed at increasing energy expenditure [63]. Furthermore, insulin receptor overexpression often occurs in DTC cells. In fact, the relative abundance of IR-A is around 40% in normal thyrocytes, while it increases to over 70% in DTC cells [64]. In addition, hyperinsulinemia determines an increase in the bioavailability of insulin-like growth factor 1 (IGF-1) through the inhibition of the synthesis of IGF binding protein 1-2 (IGFBP1-2) and the stimulation of the production of IGF-1 by the liver. The increased bioavailability of IGF-1 may contribute to tumor progression through the stimulation of IGF-1R [65]. More recently, it was demonstrated that insulin resistance may influence the evolution of thyroid nodules through an enhancement of angiogenesis and intranodular vascularization [66]. This phenomenon is probably caused by vascular endothelial growth factor (VEGF) overexpression and the consequent promotion of endothelial cell proliferation. Thus, in the condition of insulin resistance, the concomitance of hyperinsulinemia, hyperthyrotropinemia, increased bioavailability of IGF-1, and increased angiogenesis in thyroid nodules may represent important risk factors for DTC in overweight and obese patients (Figure 1).

## 7. Conclusions

In the last three decades, the incidence of thyroid cancer has increased simultaneously with the increase of obesity rates. Analyzing previous literature, growing interest has been focused on the adipocyte-secreted mediators as a sprouting factor in cancer pathophysiology. The most recent meta-analyses and reviews have demonstrated the causal role of APN and have reconstructed the signaling pathway that this and other molecules induce in obese patients with thyroid cancer [67]. The review conducted by Kim in 2017 confirmed that obesity accelerates the growth and progression of thyroid cancer, determining a shorter survival and promoting anaplastic transformation through elevated leptin level and the JAK2-STAT3 pathway [68]. Similarly, Averginos in 2019 stated that there was convincing evidence that excess body weight is associated with an increased cancer risk of at least 13 anatomic sites, including the thyroid; authors focused attention on insulin receptor role and chronic inflammation as major causes of cancer development [23].

However, Kim and colleagues [69], in a large retrospective study on 1579 cases, emphasized that, at present, the relationship between obesity and the pathological features of PTC remains controversial and there is still not agreement over many factors that could lead to interpretation bias. From this point of view, demographic characteristics such as age, sex, ethnicity, as well as smoking habits were analyzed on relatively small samples size in order to be able to identify their incidence on the obesity-tumor development relationship. Moreover, it is worth noting that BMI cannot be used as an exclusive criterion for assessing obesity, especially when it reflects the lack of specificity in centripetal obesity [70].

Finally, some authors have investigated the possible role of obesity on the outcomes of thyroid surgery; apart from a longer operative time and an increased risk of wound complications than patients with low BMI, obesity seems not to be associated with worse surgical outcomes. Pooling data from more than 18,000 patients, it is reasonable to perform thyroidectomy safely in obese patients, without expecting to manage major complications [71,72].

In conclusion, despite some contrasting results, the findings of this review suggested that obesity and excess body weight were related to an increased risk of thyroid cancer and the mechanisms that combine overweight with this cancer should be searched in the adipokines pathways, chronic inflammation onset, insulin-resistance development, and oxidative stress processes. Further studies, with a randomized and controlled design and large sample size, are needed to better address this interesting relation and overcome the confounding factors bias.

## Data Availability

No new data were created or analyzed in this study. Data sharing is not applicable to this article.
